# Supplementary material for: Magnetic Nanoprobes for Spatio-Mechanical Manipulation in Single Cells
Source: Nanomaterials (Basel). 2021 Aug 31;11(9):2267. doi: 10.3390/nano11092267 (PMC8471295; doi:10.3390/nano11092267)
Supplement: Supplementary file 1 [file nanomaterials-11-02267-s001.zip › nanomaterials-1342774-supplementary.pdf]

nanomaterials

Article

Magnetic Nanoprobes for Spatio-Mechanical Manipulation in Single Cells

Iuliia P. Novoselova [1,*,†], Andreas Neusch [1,†], Julia-Sarita Brand [1], Marius Otten [2], Mohammad Reza Safari [1,3], Nina Bartels [1], Matthias Karg [2], Michael Farle [4], Ulf Wiedwald [4] and Cornelia Monzel [1,*]

[1] Experimental Medical Physics, Heinrich-Heine University Düsseldorf, 40225 Düsseldorf, Germany; Andreas.Neusch@hhu.de (A.N.); jubra114@uni-duesseldorf.de (J.-S.B.); m.safari@fz-juelich.de (M.R.S.); Nina.Bartels@uni-duesseldorf.de (N.B.)
[2] Colloids and Nanooptics, Heinrich-Heine University Düsseldorf, 40225 Düsseldorf, Germany; marius.otten@hhu.de (M.O.); karg@hhu.de (M.K.)
[3] Peter Grünberg Institute, Electronic Properties (PGI-6), Forschungszentrum Jülich, 52425 Jülich, Germany
[4] Center for Nanointegration (CENIDE), Faculty of Physics University of Duisburg-Essen, 47057 Duisburg, Germany; michael.farle@uni-due.de (M.F.); ulf.wiedwald@uni-due.de (U.W.)
* Correspondence: Cornelia.Monzel@hhu.de (C.M.); Iuliia.Novoselova@hhu.de (I.P.N.)
† These authors contributed equally to this work.

Ferritin Purification

Sodium dodecyl sulphate–polyacrylamide gel electrophoresis (SDS-PAGE, 12%) carried out to confirm ferritin quality after each purification step can be seen in Figure S1. A standard Coomassie blue staining protocol was used. Theoretical molecular weight (MW) for mEGFP::HCF monomer is 48.7 kDa. This expected band is clearly observed in all loaded gel pockets indicating the presence of the desired mEGFP::HCF. Bright green color of the solution further confirms the correct expression of the complex containing mEGFP. Sephacryl S400 16/60 size exclusion column (SEC) equilibrated in buffer (20mM HEPES pH 8.0, 100 mM NaCl, pH 8.0) was used for the final purification step.

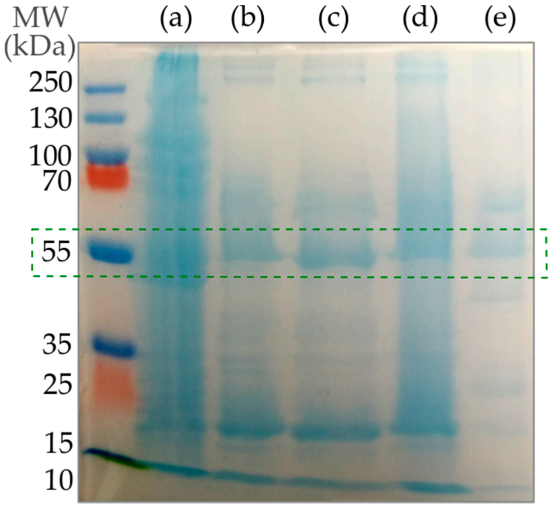

Figure S1. SDS-PAGE analysis of purification steps of ferritin shells. (**a**) directly after disrupting *E.coli* walls, (**b**) after heat denaturation at 70°C, (**c**) after 30% ammonium sulfate precipitation, (**d**) after 70% ammonium precipitation, (**e**) final product before loading on a size exclusion column (SEC).

Nanoparticles modification

Table S1. Hydrodynamic size D_H, polydispersity index (PdI), and ζ-potential for subsequent steps of magnetoferritin synthesis. Concentration c is presented after the filtering step (0.2 μm cut-off, PTFE). Stabilizing buffer (20 mM HEPES, 100 mM NaCl, pH 8.0) was used for all listed measurements. Conductivity of the medium was at 11 mS/cm for all samples while it was 0.1 mS/cm for milliQ water.

Sample	D_H, nm	PdI	ζ-Potential, mV
Non-PEGylated Ferritin Cages c = 0.5 mg/ml	15.3 ± 1.0	0.25 ± 0.00	−4.8 ± 0.7
PEGylated Ferritin Cages c = 1.0 mg/ml	20.5 ± 3.5	0.21 ± 0.01	−3.5 ± 0.7
PEGylated Magnetoferritin c = 0.7 mg/ml	39.1 ± 2.5	0.11 ± 0.01	−3.7 ± 1.2

Table S2. Hydrodynamic size D_H, polydispersity index (PdI), and ζ-potential for synomag nanoparticles with three different surface modifications. Concentration prior to filtering (0.2 µm cut-off, PTFE) was 1.0 mg/ml. Stabilizing buffer PBS (pH 7.4) was used for all listed measurements. Deviations from the ζ-potential are ascribed to the unpronounced phase plot prohibiting improvements of the final result disregarding the sub-runs increase.

Synomag	D_H, nm	PdI	ζ-Potential, mV
plain	48.1 ± 1.5	0.11 ± 0.01	−3.8 ± 0.6
NH_2	41.9 ± 0.5	0.05 ± 0.01	−1.2 ± 1.2
NH_2 - PEG_{2000}	39.1 ± 2.0	0.17 ± 0.03	−2.0 ± 2.3

Synomag colloidal stability

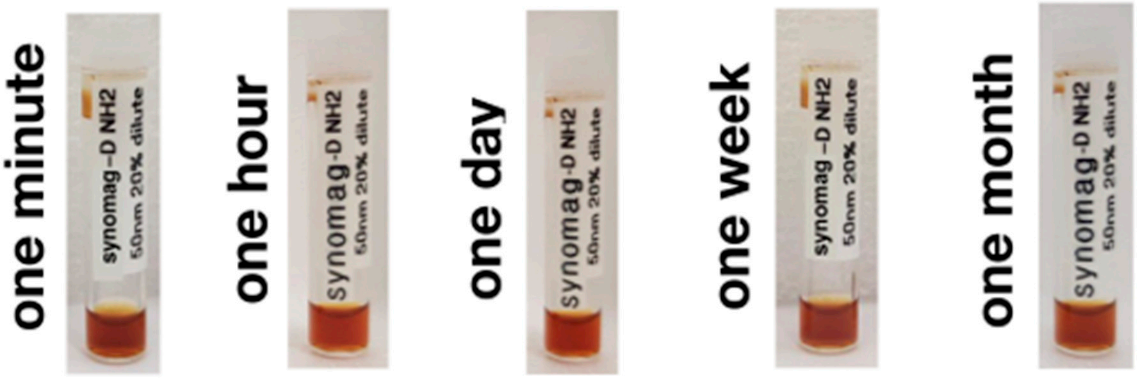

Figure S2. Qualitative assessment of the colloidal stability for synomag nanoparticles over time from 1 min up to 1 month.
